# Emotional awareness for self and others and empathic abilities in clinical depression during acute illness and recovery

**DOI:** 10.1186/s12888-024-05877-y

**Published:** 2024-07-04

**Authors:** Janine Müller, Julian Herpertz, Jacob Taylor, Thomas Suslow, Richard D. Lane, Uta-Susan Donges

**Affiliations:** 1 Department of Psychiatry, Psychotherapy and Psychosomatics, Martin Gropius Krankenhaus, Eberswalde, Germany; 2 Department of Psychiatry and Psychotherapy, Campus Charite Mitte, Universitätsmedizin, Berlin, Germany; 3 Institute for Translational Psychiatry, University of Münster, Münster, Germany; 4 David A. Dunlap Department of Astronomy and Astrophysics, University of Toronto, Toronto, Canada; 5 Department of Psychosomatic Medicine and Psychotherapy, University of Leipzig Medical Center, Leipzig, Germany; 6 Departments of Psychiatry, Psychology and Neuroscience, University of Arizona, Tucson, USA; 7 Department of Psychotherapy Science, Sigmund Freud University, Berlin, Germany

**Keywords:** Clinical depression, Emotional awareness, Empathy, German electronic levels of emotional awareness scale, Longitudinal study

## Abstract

**Background:**

The present longitudinal investigation had two major goals. First, we intended to clarify whether depressed patients are characterized by impairments of emotional awareness for the self and the other during acute illness and whether these impairments diminish in the course of an inpatient psychiatric treatment program. Previous research based on the performance measure Levels of Emotional Awareness Scale (LEAS) provided inconsistent findings concerning emotional self-awareness in clinical depression. Second, we investigated whether cognitive and affective empathic abilities change from acute illness to recovery in depressed patients.

**Methods:**

Fifty-eight depressed patients were tested on admission and after 6–8 weeks of inpatient psychiatric treatment. A sample of fifty-three healthy individuals were also examined twice at an interval of 6–8 weeks. The LEAS and the Interpersonal Reactivity Index (IRI) were administered to assess emotional awareness and empathic abilities. Written texts were digitalized and then analyzed using the electronic scoring program geLEAS, the German electronic Levels of Emotional Awareness Scale.

**Results:**

Depressed patients reported more depressive symptoms than healthy controls and less severe depressive symptomatology at time 2 compared to time 1. Independent of time, depressed individuals tended to show lower geLEAS self scores and had lower geLEAS other scores than healthy individuals. Depressed patients showed higher personal distress scores than healthy individuals at both measurement times. No group differences were observed for the cognitive empathy scales of the IRI (perspective taking and fantasy) and empathic concern, but empathic concern decreased significantly in depressed patients from time 1 to time 2. Empathic abilities as assessed by the IRI were not significantly correlated with emotional awareness for others, neither in the whole sample, nor in the patient and control subsample.

**Conclusions:**

Depressed patients seem to be characterized by impairments in emotional awareness of others during acute illness and recovery, but they also tend to show deficits in emotional self-awareness compared to healthy individuals. Self-reported cognitive empathic abilities seem to be at normal levels in depressed patients, but their heightened self-focused affective empathy may represent a vulnerability factor for depressive disorders.

**Supplementary Information:**

The online version contains supplementary material available at 10.1186/s12888-024-05877-y.

## Background

Lane and Schwartz [1] define emotional awareness as the ability to perceive and describe emotions in oneself and others. It is assumed to be an important prerequisite for successful emotion regulation [2]. The formation of emotional awareness is thought to be a process in which awareness changes from being undifferentiated into new, more sophisticated ways of representing emotions. Five hierarchically related levels of emotional awareness are distinguished: awareness of bodily sensations, action tendencies, single emotions, blends of emotions, and combinations of blends [1]. Reaching a new level modifies but does not eliminate the function of previous levels [3]. At the lowest level, emotional experience consists of somatic sensations, whereas, at the highest level, emotional experience encompasses simultaneously different blends of emotions perceived in the self and the other. Not surprisingly, high awareness of other people’s emotions was found to go along with the tendency to experience empathic responses to others [4]. Individuals tend to function at or near a consistent level of emotional awareness [5].

To assess individual differences in the complexity of emotional awareness the Levels of Emotional Awareness Scale (LEAS) was developed [6]. In the full version of the LEAS twenty emotion evoking situations are presented and respondents are asked how they and another person would feel in these situations. Subjects write down their answers in an open-ended text format. The LEAS is a performance scale. It does not rely on the subject’s assessment but measures emotional awareness directly in terms of the given response. On the basis of a wordlist and a detailed scoring manual, trained scorers assign the responses to one of the five levels for the self (self scores) and for the other person (other scores). Since its implementation, the LEAS has been administered in a series of studies, which provided strong evidence for its construct validity [7–9]. Given the time-consuming nature of manually scoring the Levels of Emotional Awareness Scale (LEAS), Barchard et al. [10] developed a computerized evaluation method that automatically scores LEAS protocols in English, streamlining the process significantly. It arrives at similar scoring results as manual scoring and was successfully implemented to score the eLEAS, the electronic version of the LEAS. Recently, the first non-English computerized assessment of the LEAS, the German electronic Levels of Emotional Awareness Scale (geLEAS), was developed and validated by Herpertz et al. [11]. Similar to the English version, its results correlate highly with those of human scorers.

Low emotional awareness can be considered as a general risk factor for psychopathology and somatic disorders [7, 12]. For example, patients with schizophrenia, posttraumatic stress disorder, and eating disorders exhibited lower scores of emotional awareness than healthy individuals [13–15]. Low emotional awareness has also been reported in patients suffering from essential hypertension and psoriasis [16, 17]. Results from clinical intervention studies show that emotional awareness can be enhanced in psychiatric and neurological patients through specific affect recognition trainings, but also through more general treatment modalities, e.g., psychoeducation, and psychotherapy [18–21].

Berthoz and colleagues [22] were the first to investigate emotional awareness in clinical depression using the LEAS. Their results indicate that acutely depressed patients are characterized by a lower awareness of their own emotions as well as a reduced awareness of other people’s emotions compared to healthy individuals. In a longitudinal intervention study, Donges et al. [23] observed that acutely depressed patients manifested lower LEAS other scores but not lower LEAS self scores than healthy individuals. After seven weeks of inpatient psychotherapeutic treatment program, depressive symptomatology decreased substantially and emotional awareness for others increased significantly in depressed patients, without reaching the emotional awareness level of healthy subjects. The authors concluded that acutely depressed individuals seem not to be impaired in the complexity of their own emotional experience, but they exhibit a reduced ability to empathize with other people. Feelings of disconnection with and distance from other people are frequent experiences in depression [24, 25].

The construct of empathy encompasses different mental processes from low-level mechanisms such as emotional contagion to high-level processes such as perspective-taking [26]. These processes can be subdivided into two major components: cognitive and affective empathy [27]. Cognitive empathy is commonly understood as the capacity for mental perspective-taking - recognizing others’ thoughts, intentions, or emotional states. In contrast, affective empathy refers to the vicarious experience of emotions, where an observer emotionally resonates with someone else’s emotional condition [27]. The abilities to empathize vary between individuals and are considered stable personality traits [28]. However, changes to one’s empathic abilities have been observed in acute phases of mental illness [29]. Findings from behavioral genetic research indicate that affective empathy could be more heritable than cognitive empathy [30].

The Interpersonal Reactivity Index is a widely used self-report instrument designed to assess four different types of empathy: perspective taking, fantasy, empathic concern, and personal distress [31]. Perspective taking refers to the tendency to adopt the psychological point of view of others. Fantasy relates to tendencies to transpose oneself imaginatively into feelings and actions of fictitious characters in books, movies, and plays. Empathic concern comprises other-oriented feelings of concern and sympathy for unfortunate others. Finally, personal distress refers to self-oriented feelings of personal unease and anxiety in tense interpersonal situations [28]. In previous studies on empathy the subscales Perspective taking and Fantasy of the IRI were frequently combined into a single cognitive empathy factor, whereas the subscales Empathic concern and Personal distress were summarized to create a single affective empathy factor [32, 33]. However, recent factor-analytic research has confirmed the four-factor model originally proposed [31] suggesting that the best practice for scoring the scale is to obtain four separate scores for the IRI [34].

In the last decades, several cross-sectional studies have investigated empathic abilities in depressed patients. As self-focus is common in clinical depression [35] it can be expected that this self-centeredness diminishes patients’ understanding towards other people’s feelings and needs. In many previous studies, acutely depressed patients have been found to be characterized by elevated levels of personal distress compared to healthy individuals [36–43] (see for a review [44]). There is also evidence that, when acutely ill, depressed patients may exhibit reduced perspective taking [40–43, 45]. Only very few of the aforementioned studies revealed alterations of empathic concern [45] or fantasy [40] in depressed patients. Recently, Dittrich et al. [46] examined empathic abilities in women remitted from major depression and revealed heightened personal distress in this sample but no alterations of empathic concern, perspective taking, or fantasy in comparison to healthy women. The latter finding suggests that increased negative emotional responses to the misfortune or suffering of others could persist after remission.

The present investigation had two major objectives. First, we examined emotional awareness in depressed patients during acute illness and recovery throughout the course of an inpatient psychiatric treatment program. To assess emotional awareness, the LEAS was administered to depressed patients and a sample of healthy individuals at two time intervals separated by 6–8 weeks. Against the background of previous findings [22, 23] it was hypothesized that when in an acute state, depressed patients would exhibit a lower emotional awareness of other persons compared with healthy individuals. We hypothesized that after six to eight weeks of treatment and a decrease in depressive symptoms, patients would show an increase of emotional awareness for others. The results of Berthoz et al. [22] and Donges et al. [23] are contradictory about deficits in awareness of own emotions in acute depression and require further investigation. Second, we compared cognitive empathic abilities (perspective taking and fantasy), and affective empathic abilities (personal distress and empathic concern) of depressed patients during acute illness and recovery with those of healthy individuals. To this aim, the IRI was also administered at the same interval. To our knowledge, our longitudinal study is the first to explore how empathic responding changes from acute illness to recovery in individuals with major depressive disorder. As mentioned above, previous research based on cross-sectional data has shown that when in an acute state of illness depressed patients exhibit primarily increased personal distress [36–41, 43] and, to a lesser extent, reduced perspective taking, fantasy, and empathic concern [40–42, 45]. Reductions in perspective taking, fantasy, and empathic concern were not observed in remitted depression [46]. Against this background, we hypothesized that depressed patients have higher personal distress scores in acute illness as well as after partial remission than healthy controls. Moreover, it was expected that from acute illness to recovery depressed patients could show an increase in perspective taking, fantasy, and empathic concern. Finally, we explored whether cognitive and affective empathic abilities are linked to emotional awareness for others. This study provides information on what kind of emotional awareness deficits and empathic abilities are observed in acute depression and whether these deficits or characteristics diminish or change during recovery.

## Methods

### Participants

The final patient sample consisted of 58 individuals (33 women), who had been admitted to the Department of Psychiatry, Psychotherapy and Psychosomatics at the Martin Gropius Krankenhaus Eberswalde (Germany). All patients fulfilled the criteria for a DSM 5 diagnosis of major depressive disorder [47]. The Structured Clinical Interview (SKID-5-CV [48]) was administered to assess psychiatric diagnoses. The interviewers were trained, instructed, and supervised by experienced psychiatrists. Ten patients were suffering from an additional anxiety disorder (i.e., social anxiety disorder, panic disorder, generalized anxiety disorder or other specified anxiety disorder (*n* = 8)) or a posttraumatic stress disorder (*n* = 2). Most patients (*n* = 53) were treated with antidepressant medication (selective serotonin reuptake inhibitors, serotonin-noradrenaline reuptake inhibitors, serotonin-norepinephrine dopamine reuptake inhibitors, noradrenaline and specific serotonergic antidepressants, or tricyclic antidepressants). Patients with current or a history of substance abuse or dependence, schizophrenia spectrum disorders, bipolar disorders, neurocognitive disorders, neurological diseases, and organic impairments were excluded. Mean duration of the index episode was 7.17 months (SD = 5.54). For twenty-nine patients the present depressive episode was the first occurrence of major depression. In our previous longitudinal study on emotional awareness in clinical depression [23], depressed patients were tested at intake and at the end of their treatment (with an average duration of 7 weeks between measurements). To ensure good comparability between the previous and our new longitudinal data we assessed patients’ emotional awareness after admission and then again after 6 to 8 weeks of treatment. In our patient sample, the mean number of days between measurement 1 and measurement 2 was 50.28 (SD: 3.75).

Four depressed patients were excluded from data analysis because they had a BDI-II score of < 14, which indicates no or minimal depressive symptoms. Twelve patients withdrew from the study after the first session. In total, sixteen patients were not part of the final patient sample.

Patients were paid a fee at the end of the study. Patients took part in a multimodal treatment program comprising individual (once a week) and group psychotherapy sessions (three times a week) in combination with music therapy, sports and arts therapy, physiotherapy, and dietetic treatment. Group psychotherapy included psychoeducation, relaxation exercises, and mindfulness training.

The final control sample included 53 healthy individuals (32 women), who had read a public notice about the study. Control participants were interviewed using the Mini-International Neuropsychiatric Interview (M.I.N.I [49]). Individuals meeting the criterion of no current or past mental disorders were included in the study. Healthy individuals were also tested twice at an interval of 6 to 8 weeks. Five control participants had to be excluded from data analysis because they had BDI-II scores of > 19 at time 1 or time 2, which indicate levels of moderate or severe depression. These five healthy participants were not part of the final control sample. All study participants were native German speakers. For study inclusion, participants had to be between 18 and 69 years of age.

### Measures

The Levels of Emotional Awareness Scale (LEAS [6]; German adaptation [50]) is a test, which comprises 20 scenarios, each described in two to four sentences and each involving two persons (e.g., scenario 2, LEAS version A: “A loved one gives you a back rub after you return from a hard day’s work. How would you feel? How would your partner feel?”). For each scenario, subjects are asked to write down their feelings and those of the other person in an open-ended response format. They are instructed to use as much or as little of the page as needed to answer the two questions. Reliable structural scoring criteria can be used to assess the degree of differentiation and integration of words denoting emotion attributed to the self and other. Participants’ responses are scored separately for each scene. Separate scores are given for the emotion described for the self and for the other (0–4) corresponding to the underlying cognitive-developmental theory of five levels of emotional awareness [1]. High scores on the LEAS reflect emotional differentiation in verbalizing emotions and awareness of emotional complexity in the self and the other. Lower scores on the LEAS, unlike many self-questionnaires on emotional awareness, are not correlated with negative affect [51, 52]. In our study, depressed and control participants were given the paper-based version of the LEAS. Two parallel versions of the scale, the LEAS A and the LEAS B were administered each containing 10 scenarios. All written texts were digitalized and then analyzed using the electronic scoring program geLEAS, the German electronic Levels of Emotional Awareness Scale, which represents an automated method for evaluating emotional awareness showing strong correlations with hand scoring [11].

The *Beck Depression Inventory* (BDI-II [53]; German adaptation [54]) is a multiple-choice self-report scale, which assesses the severity and presence of depressive symptoms such as loss of interest, hopelessness, self-blame, guilt, fatigue, and loss of appetite during the last two weeks. Respondents are asked to rate 21 items based on four response choices according to the severity of the symptoms, ranging from the absence of a symptom to an intense level. A value of 0 to 3 is assigned to each answer. Higher scores suggest more severe symptoms.

The *Interpersonal Reactivity Index* (IRI [28]; German translation [55]) consists of four subscales (perspective taking, fantasy, empathic concern, and personal distress), each of which assesses a specific aspect of empathy. Each of the subscales comprises 7 items. Items are answered on a 5-point Likert scale ranging from “Does not describe me well” [1] to “Describes me very well” [5]. The subscales Perspective taking and Fantasy assess aspects of cognitive empathy (the tendency to adopt the psychological point of view of others (perspective taking) and the tendency to imaginatively transpose oneself into the feelings and actions of fictitious characters (fantasy)). The other two subscales refer to affective empathic abilities. Empathic concern measures other-oriented feelings of sympathy and concern for unfortunate others. Personal distress assesses the negative feelings one experiences when seeing another in distress.

The *Multiple-Choice Vocabulary Intelligence Test* (Mehrfachwahl-Wortschatz-Intelligenztest, MWT-B [56]) is a performance test consisting of 37 items, which assesses verbal intelligence. Each item comprises four pronounceable pseudo-words and one real word. Subjects are asked to recognize the real word. The number of correct responses is transformed into IQ-scores using normative data.

### Procedure

Study participation consisted of an initial interview session in which inclusion criteria were assessed. At the first test session, study participants filled out the sociodemographic questionnaire and the MWT-B. Then they completed the psychological tests BDI-II, IRI, and LEAS. In each study group, approximately half of the participants were given LEAS test version A first and, at time 2, LEAS test version B. For the other participants, the order of presentation was reversed. Six to eight weeks after the first test session participants again completed the BDI-II, IRI, and LEAS.

### Statistical analyses

We used *Chi*^2^-tests and *t*-tests for independent samples to identify differences between study groups in socio-demographic variables and verbal intelligence. Levene’s test was applied to assess the equality of variances between groups. Mann-Whitney U tests were administered in analyses comparing patients with and without anxiety disorders. The geLEAS data for the self and other score were analyzed by means of 2 × 2 mixed ANOVAs with study group (depressed vs. healthy individuals) as between-subjects factor and time (test 1 vs. test 2) as within-subjects factor. To examine the time course of depressive symptoms (BDI-II), and empathic abilities (IRI) separate 2 × 2 ANOVAs with group as a between-subjects factor and time as a within-subjects factor were calculated. We applied *t*-tests for independent and dependent samples as follow-up tests. To explore the relationships of cognitive and affective empathy (IRI scales) with geLEAS scores we performed product-moment correlation analyses in the patient sample and in the healthy control sample. To increase power for the identification of relations between geLEAS and IRI we calculated additional explorative correlation analyses in the whole sample. Results were considered significant at *p* < 0.05, two-tailed. All calculations were administered using SPSS 29.0 (IBM Corp., Armonk, NY, USA).

Data of the BDI-II, geLEAS, and IRI were tested for extreme outliers as a function of study group and measurement time. A threshold of 3 standard deviations above or below the mean was used to define outliers (cf [57]). Administering this threshold two outlier values were identified in the healthy control sample: one (low) value for geLEAS other and one (low) value for IRI empathic concern. Shapiro-Wilk tests were applied to assess the normality of distribution. In the patient sample, there was a significant departure from normality for the BDI-II (time 1), *W* (58) = 0.956, *p* = 0.03, and for the BDI-II (time 2), *W* (58) = 0.923, *p* = 0.001. Similarly, there were violations of normality in the control sample for the BDI-II (time 1), *W* (53) = 0.909, *p* < 0.001, and for the BDI-II (time 2), *W* (53) = 0.876, *p* < 0.001. After outlier correction, none of the other scales (geLEAS and IRI) showed significant deviations from normality. For the BDI-II data, we calculated additional non-parametric tests to examine whether study groups differed in depressive symptoms at time 1 or time 2 (Mann-Whitney U tests) and whether depressive symptoms differed between time 1 and 2 within each study group (Wilcoxon tests for paired samples).

As previous findings suggest that affective empathy [58] and emotional awareness [59] can be increased in anxiety disorders, we explored whether presence of an anxiety disorder influenced depressed patients’ emotional awareness, empathic abilities, and depressive symptoms at time 1 or time 2. However, no significant differences were observed between patients with and those without an anxiety disorder in emotional awareness, empathic abilities, and depressive symptoms. The relevant descriptive statistics and Mann-Whitney U test results are presented in the supplementary tables S1 and S2 (see supplemental information).

A priori analyses of statistical power were conducted using the program G*Power (version 3.1.9.2.) [60]. First, a calculation based on *differences between two independent means - two groups* indicated a required sample size per group of 51 to detect a medium effect d = 0.5 with an alpha value of 0.05, one-tailed, and a power of 0.8. Note that in the longitudinal study of Donges et al. [23] the group difference between depressed and healthy individuals for the LEAS other score at time 1 had an effect size of d = 0.78. Second, a calculation with G*Power based on *repeated measures ANOVA, F tests, within–between interactions* indicated a required total sample size of 72 to detect a small to medium effect size (*f* = 0.15) given an alpha value of 0.05, a power of 0.80 (with two groups and two measurements), a correlation between repeated measures of 0.60, and a non-sphericity correction of 1.

## Results

### Sociodemographic variables and verbal intelligence

Descriptive statistics for sociodemographic and psychological characteristics of study groups are presented in Table 1. Depressed patients did not differ from healthy individuals in age, sex ratio, level of school education, and verbal intelligence (see Table 1 for details).

**Table 1:** Demographic, clinical, and intelligence test data of depressed patients and healthy individuals (means (with SD in parentheses) or frequency values)

Variable	Depressed patients (*N* = 58) Mean (SD)	Healthy individuals (*N* = 53) Mean (SD)	t / χ^2^	*p*
Age	46.17 (13.93)	44.60 (12.62)	0.62	0.54
Sex (female/male)	33/ 25	32/ 21	0.14	0.71
Level of school education *N* 8th grade *N* 9th grade *N* 10th grade *N* 11th grade *N* 12th grade	2 4 31 7 14	0 1 22 7 23	7.31	0.12
Intelligence (IQ; MWT-B)	111.29 (14.93)	114.04 (17.47)	-0.89	0.37
Total illness duration (in years)	7.07 (8.88)	-	-	-
Number of illness episodes	2.26 (1.75)	-	-	-

MWT-B: Multiple-Choice Vocabulary Test version B

### Emotional awareness for self and others: longitudinal analysis

The geLEAS scores for self and other are presented in Table 2. A 2 × 2 mixed ANOVA based on geLEAS self scores revealed a marginally significant effect of group, *F* (1, 109) = 3.17, *p* < 0.08, *ηp²* = 0.03. No other effect was significant. According to our results, depressed individuals tended to show lower geLEAS self scores compared to healthy individuals. A 2 × 2 mixed ANOVA based on geLEAS other scores yielded a significant effect of group, *F* (1, 109) = 6.06, *p* = 0.015, *ηp²* = 0.05. No further effects were observed. Independent of time, depressed patients had lower geLEAS other scores than healthy individuals. We calculated an additional 2 × 2 mixed ANOVA based on geLEAS other scores after excluding one healthy individual with an extreme outlier value. A significant effect of group was observed, *F* (1, 108) = 7.93, *p* = 0.006, *ηp²* = 0.07. No further effects were found. Thus, depressed patients had lower geLEAS other scores than healthy participants.

**Table 2:** Level of depressive symptoms, emotional awareness, and empathic abilities as a function of study groups at the two test sessions (means with SD and range in parentheses)

	Depressed patients (*N* = 58)		Healthy individuals (*N* = 53)	
	Time 1 Mean (SD and range)	Time 2 Mean (SD and range)	Time 1 Mean (SD and range)	Time 2 Mean (SD and range)
BDI-II	31.66 (9.46, 18–54)	19.43 (13.45, 2–52)	5.02 (4.20, 0–17)	4.87 (4.63, 0–17)
geLEAS Self	25.84 (6.03, 14–38)	25.21 (5.98, 12–37)	26.92 (6.06, 14–37)	27.66 (5.36, 13–38)
geLEAS Other	21.47 (6.59, 6–33)	21.93 (5.51, 11–35)	23.96 (5.96, 11–36)	24.42 (5.59, 6–37)
IRI – EC	28.48 (4.35, 18–35)	27.50 (3.98, 19–35)	27.41 (4.55, 17–35)	27.00 (5.23, 10–35)
IRI – F	19.38 (6.58, 7–33)	19.26 (5.81, 8–31)	20.13 (5.68, 11–34)	19.85 (5.39, 10–35)
IRI – PT	24.07 (5.17, 12–35)	23.81 (4.52, 12–34)	24.58 (4.61, 14–34)	24.85 (4.48, 14–34)
IRI – PD	22.97 (5.02, 11–33)	22.09 (4.26, 12–32)	17.85 (3.84, 10–27)	17.62 (3.74, 9–27)

BDI-II: Beck Depression Inventory; geLEAS: German electronic Levels of Emotional Awareness Scale; IRI - EC: Interpersonal Reactivity Index subscale Empathic concern; IRI - F: Interpersonal Reactivity Index subscale Fantasy; IRI - PT: Interpersonal Reactivity Index subscale Perspective taking; IRI - PD: Interpersonal Reactivity Index subscale Personal distress

### Number of words written in the LEAS: longitudinal analysis

The results of a 2 × 2 mixed ANOVA based on number of words written in the LEAS indicated no significant main or interaction effects. Therefore, it can be concluded that depressed patients (number of words – time 1: 55.52 (SD = 15.09), number of words – time 2: 56.29 (SD = 14.18)) did not write less in the LEAS than healthy individuals (number of words – time 1: 61.40 (SD = 17.57), number of words – time 2: 57.89 (SD = 15.42)).

### Depressive symptoms and empathic abilities: longitudinal analysis

A 2 × 2 mixed ANOVA based on BDI-II scores revealed main effects of time, *F* (1, 109) = 61.94, *p* < 0.001, *ηp²* = 0.36, and group, *F* (1, 109) = 186.77, *p* < 0.001, *ηp²* = 0.63. There was also a significant interaction time x group, *F* (1, 109) = 58.96, *p* < 0.001; *ηp²* = 0.35 (BDI-II scores are shown in Table 2). Depressed patients reported more depressive symptoms than healthy controls. However, at time 2 depressed patients had significantly lower BDI-II scores compared to time 1, *t* (57) = 8.31, *p* < 0.001, Cohen’s *d* = 1.09.

As BDI-II scores at time 1 and time 2 were not normally distributed, neither in the depressed nor in the control group, additional non-parametric tests (Mann-Whitney U tests) were conducted to determine whether there are differences in BDI-II scores between depressed patients and healthy controls at time 1 and time 2. The results indicated that healthy controls reported fewer depressive symptoms compared to depressed patients for both measurements (time 1: *Z* = -9.08, *p* < 0.001; time 2: *Z* = -6.83, *p* < 0.001). Moreover, we administered non-parametric tests (Wilcoxon tests for paired samples) to compare BDI-II scores between time 1 and time 2 within each study group. For depressed patients, BDI-II scores decreased from time 1 to time 2, *Z* = -6.02, *p* < 0.001, whereas for healthy individuals no significant BDI-II score difference was found, *Z* = -0.92, *p* = 0.36.

The IRI subscale scores are presented in Table 2. The results of a 2 × 2 mixed ANOVA on personal distress scores indicated a main effect of group, *F* (1, 109) = 40.18, *p* < 0.001; *ηp²* = 0.27. Depressed patients had higher personal distress scores than healthy individuals. The main effect of time and the interaction were not significant. A 2 × 2 mixed ANOVA based on the IRI scores of Empathic concern yielded only a main effect of time, *F* (1, 109) = 6.08, *p* < 0.05; *ηp²* = 0.05. At time 1 Empathic concern scores were higher than those at time 2. No other effects were significant. We calculated an additional 2 × 2 mixed ANOVA based on Empathic concern scores after excluding one healthy individual with an extreme outlier value. The ANOVA showed only a main effect of time, *F* (1, 108) = 5.09, *p* < 0.05; *ηp²* = 0.045.

A comparison of the Empathic concern scores at time 1 and time 2 for depressed patients showed that empathic concern was higher at time 1 than at time 2, *t* (57) = 2.36, *p* < 0.05, Cohen’s d = 0.31. Results of two 2 × 2 mixed ANOVAs based on the IRI scores of Perspective taking and Fantasy showed no significant effects. Thus, there were no group differences nor changes in perspective taking and fantasy over time.

### Relationships of empathic abilities with emotional awareness

Results from correlation analyses showed that in the whole sample there were no correlations between IRI scales and geLEAS scores (see Table 3). In the patient sample, Personal distress at time 2 was related to the geLEAS Self score at time 1. No further correlations between IRI and geLEAS were found in the patient sample (see Table 3 for details). In the healthy sample, Fantasy at time 1 was related to the geLEAS Self scores at time 1 and at time 2. No other correlations between IRI and geLEAS were observed in the control sample (see Table 3 for details).

**Table 3:** Product-moment correlations between empathic abilities (IRI) and emotional awareness for self and others (A) in the whole sample (*N* = 111), (B) in the patient sample (*N* = 58) and (C) in the healthy sample (*N* = 53)

	IRI – EC		IRI - PD		IRI - PT		IRI - F	
	Time 1	Time 2	Time 1	Time 2	Time 1	Time 2	Time 1	Time 2
**A Whole sample**								
geLEAS Self								
Time 1 Time 2	0.00 -0.09	-0.07 -0.14	0.11 -0.14	0.12 -0.14	0.10 0.05	0.04 0.02	0.16 0.06	0.14 0.05
geLEAS Other								
Time 1 Time 2	-0.03 0.00	-0.05 0.00	-0.02 -0.01	-0.04 -0.02	0.06 0.09	0.08 0.08	0.17 0.11	0.10 0.08
**B Patient sample**								
geLEAS Self								
Time 1 Time 2	0.10 -0.04	-0.05 -0.18	0.25 -0.03	0.33* -0.02	0.07 -0.05	0.04 -0.08	0.00 -0.10	0.05 -0.12
geLEAS Other								
Time 1 Time 2	0.04 -0.03	-0.06 -0.11	0.20 0.09	0.19 0.13	-0.04 0.01	0.01 -0.03	0.18 0.06	0.11 -0.03
**C Healthy sample**								
geLEAS Self								
Time 1 Time 2	-0.08 -0.11	-0.08 -0.10	0.09 -0.06	0.01 -0.07	0.11 0.16	0.01 0.09	0.35** 0.27*	0.24 0.26
geLEAS Other								
Time 1 Time 2	-0.05 0.08	-0.02 0.10	-0.09 0.15	-0.09 0.07	0.17 0.16	0.11 0.15	0.13 0.16	0.08 0.19

* *p* < 0.05, ** *p* < 0.01 (two-tailed) geLEAS: German electronic Levels of Emotional Awareness Scale; IRI - EC: Interpersonal Reactivity Index subscale Empathic concern; IRI - F: Interpersonal Reactivity Index subscale Fantasy; IRI - PD: Interpersonal Reactivity Index subscale Personal distress; IRI - PT: Interpersonal Reactivity Index subscale Perspective taking

## Discussion

In the present longitudinal study, we investigated emotional awareness and empathic abilities in depressed patients during acute illness and recovery during an inpatient psychiatric treatment program. We use the term “recovery” here to refer to a state characterized by a reduction in depressive symptoms at the group level. This decrease in depressive symptoms was determined by a significant reduction in patients’ BDI-II scores between time 1 and time 2. Confirming our hypothesis, the present data demonstrate that acutely depressed patients show reduced emotional awareness of other persons compared to healthy individuals. The effect size of this group difference (pooled across both measurements) was small to medium (Cohen’s d = 0.42). Our finding is consistent with previous reports by Berthoz et al. [22] and Donges et al. [23]. However, contrary to expectations, we found no evidence for an increase of emotional awareness for others in depressed patients over time, although their depressive symptoms diminished substantially. Thus, it seems that when recovering depressed patients still manifest decreased emotional awareness of other people. This finding fits with observations that depressed patients show deficits in the recognition of emotions in facial expressions and voices [61–63].

Our finding of stable impairments in emotional awareness for others in depression contrasts with the results of Donges et al. [23] who observed a significant increment in emotion awareness for others in depressed inpatients after (partial) symptom remission. The patients of our study had a similar level of verbal intelligence and a comparable mean duration of illness (lifetime), but they were on average fourteen years older and suffered from a more severe depressive symptomatology at both measurements (the mean BDI score was 5 points higher at both times) compared to those of Donges et al. [23]. Thus, it is possible that younger age could be a factor promoting the perception of other people’s emotions when recovering from depression. Finally, psychoanalytic-interactional group therapy as administered in the study by Donges et al. [23] might be more efficient than the present psychiatric treatment program to enhance emotional awareness of others.

According to our results, depressed patients tended to exhibit reduced awareness of their own emotions compared with healthy controls. This finding is similar to that of Berthoz et al. [22] who observed that acutely depressed patients show lower awareness of their own emotions than healthy individuals. However, the difference in emotional self-awareness between depressed and healthy persons was of large effect size (d = 1.11) in the study of Berthoz et al. [22], whereas in our study the effect size was rather small (d = 0.3). The mean age of patients was comparable between the studies. These findings contrast with those reported by Donges et al. [23] suggesting no deficits in awareness of own emotions in acutely depressed patients who were somewhat younger (and less depressed) than the patients in Berthoz et al.’s [22] and the present study. It is important to note that the reduction in emotional self-awareness (and emotional awareness for others) does not appear to stem from a general reluctance to articulate emotions of oneself and others to the situational scenarios presented in the LEAS. This is evident as there was no significant difference in the number of words employed to describe one’s own emotions and those of others between individuals with depression and healthy controls.

Overall, the available evidence suggests that in acute depression and during recovery patients could be characterized by impairments in emotional awareness of others but also of themselves. Deficits in awareness for other people might be somewhat larger than those for oneself. However, it seems that the degree of these emotional awareness deficits in clinical depression is small to moderate. Further longitudinal research is necessary to clarify whether only in case of complete remission of symptoms, emotional awareness might normalize in depressed patients. In our study, patients suffered from a long depressive episode (with a mean duration of approximately seven months) and had, on average, mild depressive symptoms after six to eight weeks of treatment. During remission, patients could still experience themselves as vulnerable and fragile and avoid paying attention to their negative affects and other people’s feelings. A challenge for future research is to determine whether lower emotional awareness could be a risk factor for depression, which is already present before onset of clinical symptoms or whether it represents a consequence of the disorder. A person who becomes clinically depressed may experience a decline in the complexity of emotional awareness [1]. The longitudinal data from Subic-Wrana et al. [20] on the effects of a multimodal psychodynamic therapy program for inpatients shows that emotional awareness as measured by the LEAS total score did not change in a group of depressed patients (mean age: 43 years) after 8–12 weeks of treatment. In this study, no healthy control group was examined and no information on subscale scores of the LEAS was provided. These results suggest that emotional awareness cannot be easily improved in clinical depression by psychotherapeutic inpatient treatments.

Our findings of tendencies of reduced emotional self-awareness in depression are in accord with clinical symptoms reported by depressed patients such as feelings of emotional numbness, or undifferentiated distress [64, 65]. Experimental research using experience sampling methodology has corroborated that individuals with clinical depression have less differentiated emotional experiences than healthy individuals [66]. Problems of depressed patients to identify and become aware of their own emotions and the emotions of others during phases of acute illness could also be related to neurocognitive deficits in the domains of attention and executive functioning, which have been frequently described in depression [67–69].

In the present study, we explored for the first time how self-reported empathic responding changes from acute illness to recovery in depressed patients. Our longitudinal results indicate that depressed patients report higher personal distress than healthy individuals at both measurement times. No group differences were observed for the empathic abilities perspective taking, fantasy, and empathic concern. Moreover, contrary to our expectation, empathic concern was found to decrease in depressed patients during the treatment program. Our data corroborate our hypothesis showing that depressed patients manifest increased personal distress in phases of acute illness [36–43] as well as in a state of remission or recovery [46]. Taken together, it appears that depressed patients compared to healthy individuals are characterized by a heightened propensity to experience self-focused discomfort in response to the suffering of others. Thus, a specific type of affective empathy is increased in acute depression and persists during recovery.

Interestingly, the second type of affective empathy, empathic concern, was found to be at normal levels but decreased significantly in depressed patients from acute illness to recovery. Our results suggest that reduction of depressive symptoms goes along with a decline in empathic concern. Depressed people frequently feel dependent on others, such as a spouse. Psychotherapeutic treatments encourage depressed patients to increase self-assertiveness and autonomy [70]. In this context, depressed patients may, to some extent, reduce empathic feelings towards others. Our data indicate no impairments in cognitive empathic abilities in clinical depression, which is largely consistent with the previous literature (36–39, 43; but see 40–42, 45 for findings of reduced perspective taking in depression). Results of a recent meta-analysis on the relationship between empathy and depression based primarily on normal samples suggest associations of depression with affective but not cognitive empathy [71]. Heightened sensitivity and strong emotional responses to other persons’ distress might result in interpersonal guilt increasing the risk of internalizing problems such as depression [72].

Self-report measures of empathy have been criticized because they appear influenced by social desirability [73] and susceptible to biases in self-perception [74]. However, subjective measures of affective empathy have been found to be positively related to objective measures such as psychophysiological empathy-related responses [75] or other laboratory assessments of empathy [76, 77]. Importantly, there is also evidence from research based on objective measures that cognitive empathic abilities are not impaired in clinical depression [39, 43] but depressed patients seem to exhibit difficulties in the affective component of empathy [43]. Despite the popularity of the Interpersonal Reactivity Index as a measure of empathy and the multi-factorial structure model of empathy underlying the IRI [31], it appears necessary and worthwhile that future clinical research on empathy follows a more complex theoretical approach. Coll et al. [78] developed a new measurement framework to characterize individual differences in empathic response, in which processes of emotion identification are differentiated from processes of affect sharing. These two processes are assumed to contribute independently to empathic responses in healthy individuals and to empathic impairments in clinical conditions. A clinical group characterized by diminished empathy due to poor emotion recognition needs a different intervention than a patient group also characterized by decreased empathy, but where this is due to reductions in affect sharing [78]. Thus, it is advisable that future studies in the field should measure the components of emotion recognition and affect sharing to improve our understanding of the mechanisms and dynamics underlying empathic response.

According to our data, cognitive and affective empathic abilities are not linked to emotional awareness for others, neither in the whole sample, nor in the patient and healthy subsample. All in all, it appears that in clinical depression (as well as in healthy individuals) self-reported cognitive and affective empathic abilities are rather independent from emotional awareness for others as assessed by the performance-based LEAS. Beyond differences in the measurement construct, unrelated method factors may create method-specific variance in the observed variables and diminish the correlations between measures [79].

A number of limitations must be acknowledged in the current study. It can be criticized that our correlation analyses were not corrected for multiple comparisons, increasing the risk of false positive results. However, even without using a more conservative alpha level we observed no correlations between the geLEAS other score and the empathy scales of the IRI (neither in the subsamples nor in the whole sample). In the context of our main research questions (the longitudinal analyses of emotional awareness for the *self* and the *other* and the four empathic abilities assessed by the IRI), we calculated six 2 × 2 ANOVAs. When correcting the alpha level in these ANOVAs to *p* = 0.0083 (0.05/6) the main effect of group would be still significant for the geLEAS other scores (after excluding one healthy individual with an extreme outlier value). The effect of group would also remain significant for personal distress. However, when using the corrected alpha, the effect of time would be no longer significant for empathic concern. Thus, the latter effect should be interpreted with particular caution. Another limitation of our study is that we did not register the precise number of days between first and second administration of the LEAS (and the IRI) in the healthy control group. As a consequence, we could not use *number of days* between LEAS measurements as a covariate in comparisons between study groups. It can also be criticized that the longitudinal evaluation of participants’ depressive symptoms was performed exclusively as self-assessment (using the BDI-II). It would be methodologically stronger to combine self-report with clinician-rated instruments such as the Montgomery-Asberg Depression Rating Scale (MADRS [80]), to measure level of depressive symptoms.

Taken together, the present findings suggest that acutely depressed patients show reduced emotional awareness of other persons compared to healthy individuals and that these reductions remain stable during recovery. Moreover, our results indicate that depressed patients are characterized by increased self-focused affective empathy in acute illness as well as during recovery and manifest no differences in other-focused affective empathy and cognitive empathy compared to healthy individuals. As heightened personal distress was also found in remitted depressed patients [46] and associations of depressive symptoms with affective (but not cognitive) empathic abilities have been documented in healthy samples [71], a disposition toward high personal distress could function as a vulnerability factor for depressive disorders.

Our results can have some clinical implications. Impairments in emotional self-awareness in acutely depressed and recovering patients seem, on average, rather small. This means that for a number of depressed patients there could be no indication for trainings improving self-oriented emotional awareness. However, it appears advisable to identify those patients characterized by poor emotional self-awareness (e.g., using the geLEAS) and to offer them trainings promoting their emotional awareness [18, 19]. Overall, our findings indicate a greater potential need to improve emotional awareness for others than for one-self in depressed patients. However, it is remarkable that in our study we found evidence for a reduction in patients’ empathic concern, i.e., their other-oriented affective empathy, during treatment. During recovery, depressed patients could distance themselves, to some extent, from other people to strengthen their autonomy and protect their own perspectives and interests. These tendencies to distance themselves from others may interfere with therapeutic efforts to enhance emotion perception in other people.

### Electronic supplementary material

Below is the link to the electronic supplementary material.

Supplementary Material 1

## Data Availability

The datasets used and/or analyzed during the current study are available from the corresponding author on reasonable request.

## Abbreviations

BDI: II Beck Depression Inventory
geLEAS: The German electronic Levels of Emotional Awareness Scale
IRI: Interpersonal Reactivity Index
LEAS: Levels of Emotional Awareness Scale
MWT: B Multiple-choice Vocabulary Test version b
