# Supplementary material for: Emotional awareness for self and others and empathic abilities in clinical depression during acute illness and recovery
Source: BMC Psychiatry. 2024 Jul 4;24:488. doi: 10.1186/s12888-024-05877-y (PMC11225305; doi:10.1186/s12888-024-05877-y)
Supplement: Supplementary file 1 — Supplementary Material 1 [file 12888_2024_5877_MOESM1_ESM.docx]

**Supplementary Table S1**

Level of depressive symptoms, emotional awareness, and empathic abilities in depressed patients with and without anxiety disorders at the two test sessions (means with SD in parentheses).

|  | **Depressed patients**  **with anxiety disorders**  (N = 8) | | **Depressed patients**  **without anxiety disorders**  (N = 50) | |
| --- | --- | --- | --- | --- |
|  | Time 1  Mean (SD) | Time 2  Mean (SD) | Time 1  Mean (SD) | Time 2  Mean (SD) |
| BDI-II | 28.87 (7.62) | 18.75 (11.40) | 32.10 (9.71) | 19.54 (13.85) |
| geLEAS Self | 27.88 (4.79) | 27.88 (6.38) | 25.52 (6.18) | 24.78 (5.86) |
| geLEAS Other | 23.75 (5.47) | 25.13 (8.06) | 21.10 (6.73) | 21.42 (4.92) |
| IRI – EC | 28.50 (3.42) | 27.75 (2.92) | 28.44 (4.44) | 27.46 (4.15) |
| IRI – F | 21.12 (7.85) | 18.37 (6.78) | 19.10 (6.41) | 19.40 (5.70) |
| IRI – PT | 25.12 (6.51) | 24.75 (6.06) | 23.90 (4.99) | 23.66 (4.28) |
| IRI – PD | 23.00 (4.57) | 24.12 (3.68) | 22.96 (5.13) | 21.76 (4.29) |

BDI-II: Beck Depression Inventory; geLEAS: German electronic Levels of Emotional Awareness Scale; IRI - EC: Interpersonal Reactivity Index subscale Empathic concern; IRI - F: Interpersonal Reactivity Index subscale Fantasy; IRI - PT: Interpersonal Reactivity Index subscale Perspective taking; IRI - PD: Interpersonal Reactivity Index subscale Personal distress.

**Supplementary Table S2**

Presentation of Mann-Whitney U test results for level of depressive symptoms, emotional awareness, and empathic abilities at the two test sessions: comparisons between depressed patients with and without anxiety disorder.

|  | *U* | *Z* | *p* (two-tailed) |
| --- | --- | --- | --- |
| BDI-II Time 1  Time 2 | 165.000  197.500 | -0.79  -0.06 | .44  .96 |
| geLEAS Self Time 1  Time 2 | 153.500  145.000 | -1.05  -1.24 | .30  .22 |
| geLEAS Other Time 1  Time 2 | 159.500  148.000 | -0.91  -1.17 | .37  .25 |
| IRI – EC Time 1  Time 2 | 197.000  191.000 | -0.07  -0.20 | .96  .85 |
| IRI – F Time 1  Time 2 | 173.000  187.500 | -0.61  -0.28 | .56  .78 |
| IRI – PT Time 1  Time 2 | 169.500  192.500 | -0.69  -0.17 | .50  .87 |
| IRI – PD Time 1  Time 2 | 189.000  140.500 | -0.25  -1.35 | .82  .18 |

BDI-II: Beck Depression Inventory; geLEAS: German electronic Levels of Emotional Awareness Scale; IRI - EC: Interpersonal Reactivity Index subscale Empathic concern; IRI - F: Interpersonal Reactivity Index subscale Fantasy; IRI - PT: Interpersonal Reactivity Index subscale Perspective taking; IRI - PD: Interpersonal Reactivity Index subscale Personal distress.
